# Characterization of Red Dragon Fruit Wine Fermented with a Newly Identified Yeast Strain *Saccharomyces cerevisiae* M7

**DOI:** 10.17113/ftb.63.01.25.8784

**Published:** 2025-03

**Authors:** Tran Thanh Quynh Anh, Nguyen Tien An, Do Thi Bich Thuy

**Affiliations:** 1 Faculty of Engineering and Food Technology, Hue University of Agriculture and Forestry, Hue University, 102 Phung Hung, 49116 Hue, Vietnam; 2 Faculty of Agriculture and Forestry, Dalat University, 01 Phu Dong Thien Vuong, 66106 Dalat, Vietnam; 3 Institute of Research and Development, Duy Tan University, 254 Nguyen Van Linh, 50312 Da Nang, Vietnam; 4 School of Engineering and Technology, Duy Tan University, 254 Nguyen Van Linh, 50312 Da Nang, Vietnam

**Keywords:** *Saccharomyces cerevisiae*, yeast, wine, red dragon fruit, anthocyanins

## Abstract

**Research background:**

Dragon fruit (*Hylocereus* spp.) has been known to be a rich source of bioactive compounds, such as anthocyanins, betacyanin, betaxanthin and other phenolic substances, and it has a nutritional profile suitable to produce wine with functional properties. The aim of this study is to characterize the wine fermentation from red dragon fruit juice by a newly identified yeast strain.

**Experimental approach:**

Yeast strains from banh men, a Vietnamese traditional alcoholic fermentation starter, were screened for ethanol production using thermally pretreated red dragon fruit juice. The most potent candidate was identified by the DNA sequencing method and subjected to an optimization study using a one-factor-at-a-time approach to optimize the conditions for red dragon fruit wine fermentation.

**Results and conclusions:**

Results showed that thermal pretreatment of the red dragon fruit juice at 70 °C for 10 min resulted in a higher amount of phenols and antioxidants than at other pretreatment temperatures. Among the four isolates, M7 was the strongest alcohol fermenter, which was then identified as *Saccharomyces cerevisiae* using a DNA sequencing method. The optimal conditions for wine fermentation from red dragon fruit juice by *S. cerevisiae* M7 included a pitching rate of 10^8^ CFU/mL, an initial sucrose content of 18 % (*m*/*V*), an initial pH=4.5, fermentation temperature of 30 °C and a fermentation time of 6 days. Under these conditions, the wine fermented by *S. cerevisiae* M7 had an ethanol volume fraction of (12.1±0.2) %, the concentration of total phenolics expressed as gallic acid equivalents (37.8±0.4), anthocyanins expressed as cyanidin 3-glucoside equivalents (11.2±0.3), betacyanin (65.2±0.8) and betaxanthin (60.5±1.3) mg/L and antioxidant activity measured by DPPH scavenging capacity of (65.4±0.4) %.

**Novelty and scientific contribution:**

This study used a novel yeast strain *Saccharomyces cerevisiae* M7 for fermentation. In addition, the results of the study provide new data such as the optimal parameters and the accumulation of bioactive compounds (phenols, anthocyanins and betalains) related to the fermentation of red dragon fruit wine.

## INTRODUCTION

Wine is traditionally made from the fermentation of grape must. However, making wine from fruits other than grapes has gained much interest due to its multiple properties, such as colour, flavour and nutritional values (*1*). Many fruits from temperate regions (*e.g*. apples and berries) and tropical regions (*e.g*. banana, mango, pineapple and sweet potatoes) can be used to produce wines. As such, wines made from cherries, raspberries and blueberries contain a significant amount of polyphenols, flavonoids and anthocyanins that give the wines remarkable antioxidant, anti-proliferative, anti-inflammatory and anti-ageing capacities (*2*–*4*). Among the fruits from tropical and subtropical regions, the dragon fruit (*Hylocereus* spp.) has suitable properties for the production of wine, which is shown by the fact that the pH of the juice is between 4.3 and 5, depending on the cultivar, while glucose and fructose in the juice are between 49.1 and 104 g/L and 19.2 and 29 g/L, respectively (*5*). The red dragon fruit is also a rich source of flavonoids, betacyanin and betaxanthin, and carotene (*6*, *7*), making wine produced from these fruits a potential functional food.

During the production of fermented beverages, the yeast *Saccharomyces cerevisiae* plays an essential role in converting carbohydrates to ethanol and participating in secondary fermentation, which affects flavour and aroma development (*8*). Therefore, *S. cerevisiae* has been used as a starter culture for various fermented beverages, such as wine, whisky, cognac, sake and beer (*9*). In Vietnam, however, banh men has been used for centuries as a starter to produce traditional alcoholic beverages from rice. Banh men is produced from uncooked rice dough and oriental herbs inoculated with a starter from the previous batch (*10*). According to Lee and Fujio (*11*), banh men is similar to fermentation starters traditionally used in other Asian countries in terms of microbial composition. Among the microflora present in banh men, *S. cerevisiae* strains were reported as the main ethanol producer (*10*). In this study, we aim to screen the *S. cerevisiae* strains from banh men capable of fermenting red dragon fruit juice into wine and to investigate the effect of fermentation conditions on the quality of wine produced by the most potent strain.

## MATERIALS AND METHODS

### Chemicals

All reagents used in the experiments such as glucose, sucrose, peptone, yeast extract, Hansen broth, Hansen agar, buffer, gallic acid, cyanidin 3-glucoside, Folin-Ciocalteu’s phenol reagent, cyanidin-3-glucoside, phenol, chloroform, isoamyl alcohol, ethanol, agarose, Na_2_CO_3_, KCl, CH_3_COONa and 2,2-diphenyl-1-picrylhydrazyl (DPPH) were purchased from Sigma-Aldrich, Merck, St. Louis, MO, USA.

### Isolation of yeasts

A mass of 1 g of banh men was finely ground and suspended in 9 mL of saline. The suspension was further serially diluted with saline and spread onto Hansen agar plates. The plates were then incubated at 28 to 32 °C for 24 to 48 h until isolated colonies were obtained. The colonies were subsequently transferred to the new Hansen agar including (in %, *m*/*V*): glucose 2, peptone 1, yeast extract 0.1 and agar 1.5. The colonies that were round, smooth and white to whitish cream and the cells that had an oval shape and budding characteristics were suspected to be yeast and used for further studies.

### Red dragon fruit juice preparation

The red dragon fruits were washed under running water for about 1 min and drained for 30 min. The fruits were then peeled and the juice was extracted from the fruit pulp using an electric slow juicer (H200; Hurom, Gimhae-si, South Korea). The juice had total soluble solids of (12.5±0.3) % and a pH=4.53±0.12. The concentrations of total phenolics expressed as gallic acid equivalents (GAE), anthocyanins expressed as cyanidin 3-glucoside equivalents (CGE), betacyanin and betaxanthin of the juice were (23.11±0.69), (5.09±0.42), (31.21±0.56) and (23.12±0.39) mg/L, respectively.

### Thermal treatment of dragon fruit juice and screening of yeast strains

The dragon fruit juice was thermally treated at 60, 70 and 80 °C for 10 min, followed by cooling to ambient temperature. Sucrose was added to obtain a total soluble solid of 18 % (*m*/*V*). The yeast isolates were then inoculated into the juice at 10^7^ CFU/mL density and kept at 25 °C for fermentation. When the formation of bubbles ceased, as an indicator of the completion of primary fermentation, different quality attributes of the fermented juice samples were analyzed. The thermal treatment regimen and yeast isolate that yielded the highest amounts of ethanol, anthocyanins, total phenolic compounds, betacyanin and betaxanthin and antioxidant activity were selected for further analyses.

### Identification of selected yeast strain

The yeast isolate that produced the wine with the highest contents of ethanol and antioxidant substances was identified by internal transcribed spacer regions (ITS) sequencing method (*12*). The ITS sequence was compared against accession numbers available in the GenBank database using the Basic Local Alignment Search Tool (BLAST) (*13*).

### Total genomic DNA extraction

The selected yeast isolate was grown in Hansen broth for 24 h, followed by centrifugation at 8000×*g* (Sigma 1-16; Sigma, Osterode am Harz, Germany) for 2 min to obtain biomass. The biomass was washed with 700 μL of sterile distilled water. The genomic DNA of the yeast was extracted using the TopPURE^®^ Genomic DNA extraction kit (ABT Biomedical Solutions, Ho Chi Minh City, Vietnam) following the manufacturer's instructions. Briefly, an aliquot of 800 μL lysis buffer was added to the tube containing the biomass, mixed and 40 μL of 20 % (*m*/*V*) SDS were added. The mixture was vortexed for 2 min and incubated at 65 °C for 30 min, followed by centrifugation at 10 000×*g* for 15 min at 4 °C (Sigma 1-16; Sigma). An equivalent volume of phenol/chloroform/isoamyl alcohol (25:24:1) was added to the collected supernatant, mixed and centrifuged at 10 000×*g* for 15 min at 4 °C (Sigma 1-16; Sigma). The top layer was collected, mixed with an equivalent volume of isopropanol and incubated at –40 °C for 2 h. The mixture was then centrifuged at 10 000×*g* for 15 min at 4 °C (Sigma 1-16; Sigma) to collect genomic DNA. The genomic DNA was washed twice with 500 μL of 70 % (*m*/*V*) ethanol and resuspended in 30 μL of sterile water. The quality of the genomic DNA was verified by electrophoresis on 1 % agarose gel. An aliquot of 1 μL of RNase (100 μg/μL) was added to the DNA solution to eliminate RNA.

### DNA amplification and sequencing analysis

The genomic DNA was amplified using yeast universal primers ITS1 (5’TCCGTAGGTGAACCTGCGG 3’) and ITS4 (5’TCCTCCGCTTATTGATATGC 3’). The PCR reaction volume was 60 μL consisting of 30 μL of 2X GoTaq Green Master Mix, 3 μL of each primer (10 pmol/μL), 6 μL of genomic DNA and 18 μL of H_2_O. The polymerase chain reaction (PCR) was carried out under the following conditions: initial denaturation at 95 °C for 5 min; 30 cycles of denaturation at 95 °C for 1 min, annealing at 53 °C for 1 min and extension at 72 °C for 1 min; and final extension at 72 °C for 10 min. The PCR product was checked by electrophoresis at 70 V for 30 min on 1 % agarose gel, stained with SafeView Classic Nucleic Acid Stains (Thermo Fisher Scientific, Waltham, MA, USA). The DNA bands on the gel were visualized using Ultra Slim LED Illuminator and the size of the DNA was estimated using GeneRuler 1kb DNA Ladder (Thermo Fisher Scientific). The PCR product was sent to 1st BASE (Thermo Fisher Scientific) for DNA sequencing. The obtained sequences were then aligned and compared with sequences of species available in the NCBI database using BLAST (*13*).

### Dragon fruit wine fermentation by selected strain

The effects of fermentation conditions on the quality of fermented dragon juice were evaluated by varying several parameters one by one. The parameters of interest investigated in this study were in the following chronological order: pitching rate (10^5^–10^9^ CFU/mL), initial total soluble solids (12–21 %), initial pH (3.5–5.5), fermentation temperature (20–35 °C) and fermentation time (1–7 day). To determine the quality of the wine and the efficacy of the yeast strain, ethanol concentration, anthocyanins, phenolic content and antioxidant activity were evaluated.

### Wine quality analysis

#### Ethanol content

The ethanol content of the fermented juice was determined according to AOAC method 920.57 (*14*). Briefly, ethanol from 200 mL of the fermented juice was separated by distillation. A hydrometer was used to measure the gravity of the distillates, which were then used to calculate the ethanol volume fraction.

#### Total phenolic content

An aliquot of wine was centrifuged at 6000×*g* (Sigma 1-16; Sigma) for 15 min and used to determine the total phenolic content and antioxidant activity. The total phenolic content was determined according to the method of Singleton and Rossi (*15*). Briefly, 200 μL of the extract were mixed with 1 mL of 10 % *m/V* Folin-Ciocalteu’s phenol reagent and 1.2 mL of 10 % Na_2_CO_3_ solution. The mixture was then allowed to react for 2 h at room temperature and the absorbance was measured at 760 nm. Gallic acid was used as a standard and the total phenolic content was expressed as mg of GAE per mL of the juice.

#### Total anthocyanin content

The total anthocyanin content was determined using the pH differential method described by Lee *et al.* (*16*). Briefly, the absorbance of samples diluted in 0.025 M potassium chloride buffer (pH=1) or 0.4 M sodium acetate buffer (pH=4.5) was measured concurrently at 520 and 700 nm after 20 min of incubation at ambient temperature. The total anthocyanin content was calculated in mg of CGE per L of the juice using the following equation:


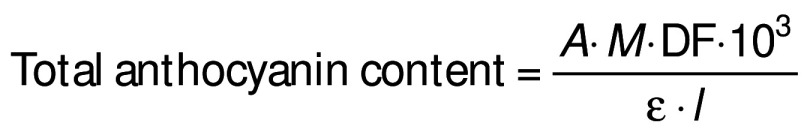
 /1/

where 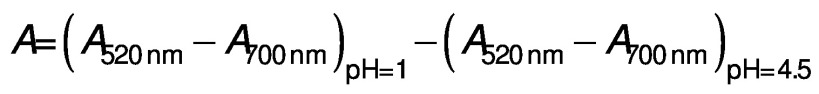 /2/

*A*_520 nm_ and *A*_700 nm_ is the absorbance of the samples at 520 and 700 nm, *M* is the molecular mass of cyanidin-3-glucoside (449.2 g/mol), DF is the dilution factor, 10^3^ is the coefficient for conversion from gram to milligram, *ε* is the molar absorption coefficient of cyanidin-3-glucoside (26 900 L/(mol·cm)) and *l* is the path length (1 cm).

#### Antioxidant activity

Antioxidant activity was determined by the DPPH radical scavenging method (*17*). Briefly, 0.4 mL of the sample or blank (ethanol) was mixed with 3.6 mL of 0.1 mM DPPH. The reaction was carried out in the dark at room temperature for 1 h. The absorbance of the resulting solution was measured at 517 nm and used to calculate the antioxidant activity of the juice as the DPPH scavenging capacity (%) using the following equation:


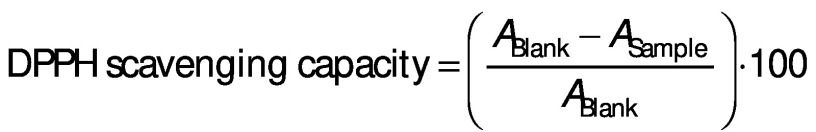
 /3/

where *A*_Blank_ is the absorbance of blank and *A*_Sample_ is the absorbance of the sample.

### Betalain content

The betalain content was quantified as described previously (*18*). Briefly, the absorbance of the samples was measured at 480 and 540 nm to determine betacyanin and betaxanthin contents, respectively. The contents of each betalain compound (mg/mL) were calculated using the following equations:


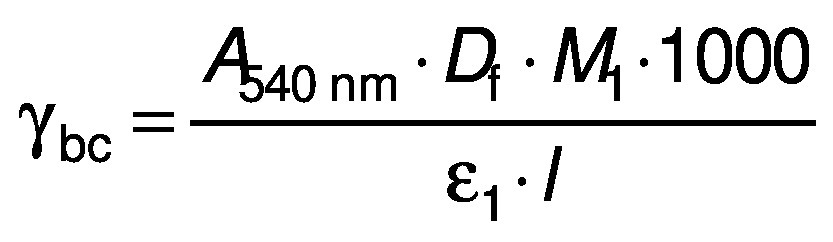
 /4/


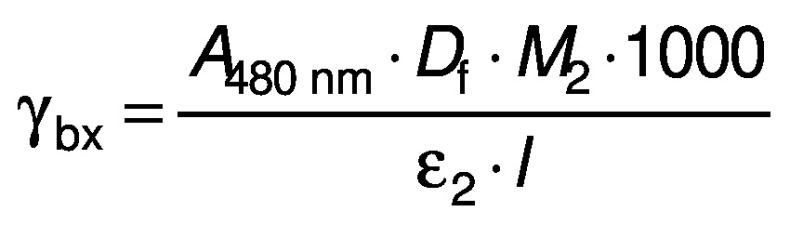
 /5/

where *γ*_bc_ and *γ*_bx_ are the betacyanin and betaxanthin mass concentration (mg/L), respectively, *A*_540 nm_ and *A*_480 nm_ are the absorbance of the samples at 540 and 480 nm, respectively, *M*_1_ and *M*_2_ are the betaxanthin (308 g/mol) and betacyanin (550 g/mol) molecular mass, respectively, *ε*_1_ and *ε*_2_ are the molar absorption coefficient of betaxanthin (48000 L/mol) and betacyanin (6000 L/mol), respectively, *D*_f_ is the dilution factor, 1000 is the coefficient for conversion from gram to milligram, and *l* is the path length (1 cm).

### Statistical analysis

Data were reported as mean value±S.D. of triplicate experiments. One-way ANOVA, followed by Duncan’s test, was used to determine the difference between the means at a significance level of 0.05. Statistical analyses were performed using SPSS v. 17.0 software (*19*).

## RESULTS AND DISCUSSION

### Isolation and screening of yeast strains

Among 32 isolated colonies obtained from Hansen agar plates, 18 isolates (designated as strains M1 to M18) were classified as yeasts based on their cell morphology observed using electron microscopy. These isolates were screened for ethanol production by fermenting red dragon juice at 28 °C for 24 h. The results showed that four isolates (M2, M7, M11 and M17) produced ethanol (Table S1). These four strains were selected for further experiments.

### Effect of thermal pretreatment and yeast isolates on the quality of wine

Temperature is one of the most important factors affecting the extraction of bioactive compounds from biomass (*20*). Thus, the thermal pretreatment of dragon fruit juice in this study was expected to affect the quality of the wine in terms of bioactive components, such as phenols, anthocyanins, betacyanin, betaxanthin and their antioxidant activity.

On the other hand, ethanol content is another key element that determines the yield and activity of bioactive compounds (*21*). During fermentation, yeasts continuously produce ethanol, which changes the ethanol content of the fermented juice, hence the extraction of bioactive compounds. In this experiment, we investigated how yeast isolates from banh men affect the quality characteristics of dragon fruit wine made from juice that was thermally pretreated at different temperatures. The two-way ANOVA showed that thermal pretreatment and yeast isolates and the interaction between these two factors affected the amounts of ethanol, total phenolic, anthocyanin, betacyanin and betaxanthin content and antioxidant activity of dragon fruit wine (p<0.05). Thus, the yeast isolates produced higher amounts of ethanol at a pretreatment temperature of 70 °C than at 60 or 80 °C (Table 1). The concentrations of phenolic compounds, bioactive compounds (anthocyanins, betacyanin and betaxanthin) and antioxidant activity of the wine produced from the juice pretreated at 70 °C were also significantly higher than at the other temperatures. This result indicates that the phenolic compounds were most effectively extracted from the dragon fruit pulp particles into the juice at 70 °C. The results of this experiment were consistent with a study by El Darra *et al*. (*22*), in which thermovinification pretreatment at 70 °C promoted the release of phenolic compounds from grape skin cells. Among the isolates capable of ethanol fermentation, M7 yielded higher volume fractions of ethanol ((6.3±0.3) % than M2, M11 and M17 at a pretreatment temperature of 70 °C. In addition, the concentrations of total phenolics as GAE (27.2±5.4), anthocyanins as CGE (10.0±0.2), betacyanin (50.4±1.0) and betaxanthin (43.6±0.7) mg/L and DPPH scavenging capacity (57.6±0.4) % of the juice pretreated at 70 °C and fermented by M7 were significantly higher than by other isolates. In short, the results of this study show that the pretreatment temperature of 70 °C and the use of isolate M7 appear to be the most suitable for the production of wine from red dragon fruit juice and were therefore selected for further experiments.

**Table 1:** Effect of thermal pretreatment and yeast isolates on the quality of dragon fruit wine

Pretreatment temperature/°C	Yeast isolate	*φ*(ethanol)/%	Total phenolic as *w*(GAE)/(mg/mL)	Anthocyanin as *w*(CGE)/(mg/L)	DPPH radical scavenging activity/%	*γ*(betacyanin)/(mg/L)	*γ*(betaxanthin)/(mg/L)
60	M2	(4.0±0.1)^ef^	(14.8±2.1)^cde^	(3.6±0.4)^g^	(38.1±0.5)^ef^	(26.9±1.1)^f^	(26.4±0.6)^g^
	M7	(5.2±0.4)^bcd^	(13.4±1.3)^def^	(6.7±0.4)^cde^	(42.4±0.6)^c^	(46.6±0.9)^b^	(38.5±0.6)^c^
	M11	(5.1±0.4)^bcd^	(19.0±2.2)^bcd^	(5.6±0.5)^ef^	(39.4±0.6)^de^	(44.7±0.9)^b^	(35.5±0.8)^de^
	M17	(4.4±0.2)^def^	(12.1±1.8)^ef^	(5.9±0.4)^def^	(28.1±0.1)^i^	(35.4±0.8)^de^	(30.6±1.0)^f^
70	M2	(4.7±0.3)^cde^	(22.7±1.1)^ab^	(4.9±0.5)^f^	(46.1±0.4)^b^	(26.64±1.0)^f^	(28.4±0.7)^fg^
	M7	(6.3±0.3)^a^	(27.2±5.4)^a^	(10.0±0.2)^a^	(57.6±0.4)^a^	(50.4±1.0)^a^	(43.6±0.7)^a^
	M11	(5.6±0.2)^abc^	(20.3±1.6)^bc^	(7.46±0.40)^bc^	(40.4±0.7)^d^	(46.2±0.5)^b^	(39.1±0.9)^b^
	M17	(5.0±0.2)^bcd^	(20.6±1.2)^bc^	(6.9±0.4)^cde^	(35.6±0.6)^fg^	(44.5±0.8)^b^	(36.5±1.2)^cde^
80	M2	(4.0±0.2)^ef^	(16.5±1.4)^bcde^	(5.0±0.4)^f^	(30.7±1.0)^h^	(28.3± 0.9)^f^	(28.3±0.9)^fg^
	M7	(5.7±0.4)^ab^	(15.6±1.2)^cde^	(8.6±0.6)^b^	(42.8±0.7)^c^	(41.4±0.5)^c^	(41.4±0.5)^ab^
	M11	(4.6±0.4)^cde^	(11.8±1.2)^ef^	(6.9±0.3)^cd^	(35.3±0.3)^g^	(37.7±0.6)^d^	(37.7±0.6)^cd^
	M17	(3.6±0.2)^f^	(8.9±1.2)^f^	(6.5±0.5)^cde^	(24.2±0.6)^j^	(35.0±0.7)^e^	(35.0±0.7)^e^

GAE=gallic acid equivalent, CGE=cyanidin-3-glucoside equivalent. Results are mean values of triplicate analyses±standard deviation. Mean values in the same column without a common letter differ significantly, p≤0.05

### Identification of yeast strain

The BLAST (*13*) search of the ITS sequence of M7 isolate against reference sequences of *Saccharomyces cerevisiae* species from GenBank (accession numbers of the ITS sequences are KY109257.1 and MZ452353.1) showed a high similarity (>99 %), indicating that M7 isolate belongs to this species (Table 2). Thus, the strain was named *S. cerevisiae* M7.

**Table 2:** Results of the identification of M7 strain

Yeast strain No.	Accession numbers of the ITS sequences	Species	Similarity/%
M7	KY109257.1	*Saccharomyces cerevisiae*	100
	MZ452353.1	*Saccharomyces cerevisiae*	99

### Optimization of red dragon juice fermentation by the yeast strain

#### Effect of pitching rate

**Fig. 1:**
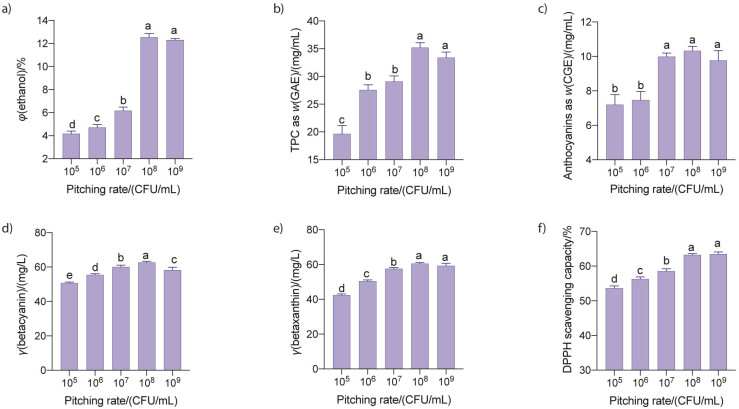
Effect of pitching rate on: a) ethanol content, b) total phenolic, c) total anthocyanin, d) betacyanin and e) betaxanthin mass concentrations, and f) antioxidant activity of the red dragon fruit wine. The juice, with a total soluble solid content of 18 % (*m*/*V*) and pH=4.5, was fermented by S. *cerevisiae* M7 at different pitching rates for 7 days at 25 °C. Data are mean values of triplicate analyses±standard deviation. Mean values in each graph without a common letter differ significantly (p≤0.05). GAE=gallic acid equivalent, CGE=cyanidin-3-glucoside equivalent

Studies have shown that pitching rate is a crucial factor affecting the wine fermentation from different fruit juices by yeasts (*23*, *24*). In this study, the effect of pitching rate on ethanol production, total phenolic and anthocyanin contents, and antioxidant activity of the dragon fruit wine was investigated. Results show that the ethanol volume fraction and concentrations of phenols determined as GAE, anthocyanins determined as CGE, betacyanin and betaxanthin and DPPH scavenging capacity of the fermented juice increased with the increase in pitching rate and reached the maximum values of (12.5±0.3) %, (35.2±0.9) mg/mL, (9.9±0.2) mg/L, (62.6±0.7) mg/L, (60.5±0.7) mg/L and (63.2±0.3) %, respectively, at the pitching rate of 10^8^ CFU/mL, then levelled off with further increment of pitching rate (Fig. 1). The increase in ethanol production in response to the increase of inoculum size to a certain level was also observed in a study by Huan *et al*. (*25*), who reported that the maximum volume fraction of ethanol of around 3.5 % was obtained at a yeast rate of *φ*=2 % after 40–48 h of fermentation, while lower or higher amount of yeast added to the juice resulted in lower ethanol productivity. Similarly, Samson *et al.* (*26*) also documented that ethanol production in pomegranate fruit juice fermentation increased with the increase in the pitching rate from 2 to 8 %, then decreased at higher inoculum sizes. In another study of wine produced from cactus pear and lantana camara fruit juice, a 10 % of yeast inoculation, compared with 8 or 12 %, was found to be most favourable for wine fermentation (*27*). In the current study, the increase in the concentration of anthocyanin and phenolic compounds and antioxidant activity of the red dragon wine at a higher pitching rate (*e.g.* 10^8^ CFU/mL) was likely to be the result of the higher ethanol volume fraction obtained at this pitching rate, which enhanced the extraction of these substances from the red dragon fruit pulp particles.

#### Effect of initial sucrose content

**Fig. 2:**
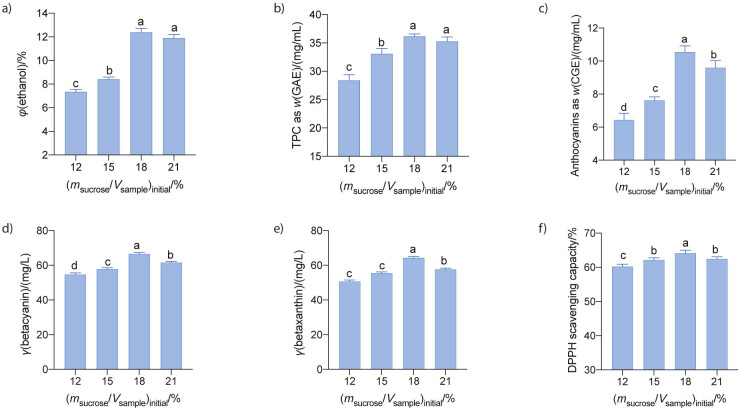
Effect of initial sugar content on: a) ethanol content, b) total phenolic, c) total anthocyanin, d) betacyanin and e) betaxanthin mass concentrations, and f) antioxidant activity of the red dragon fruit wine. The juice with different initial sugar content and a pH=4.5 was fermented by *S. cerevisiae* M7 at a pitching rate of 10^8^ CFU/mL and 25 °C for 7 days. Data are a mean value of triplicate analyses±standard deviation. Mean values in each graph without a common letter differ significantly, p≤0.05. GAE=gallic acid equivalent, CGE=cyanidin-3-glucoside equivalent

As one of the most important carbon sources for wine fermentation by yeasts (*9*), sucrose has been shown to be the most influential factor affecting the quality of wine produced from fruit juice, compared to other factors such as SO_2_ treatment, yeast inoculation and fermentation time (*24*). In this study, we investigated the fermentation of red dragon fruit wine using *S. cerevisiae* M7 by varying the initial sucrose content from 12 to 21 % (*m/V*) while the pitching rate and the juice pH were kept constant at 10^8^ CFU/mL and 4.5, respectively. Results showed that higher initial sucrose content led to higher ethanol production and the maximum ethanol yield (12.3±0.3) % was obtained at the initial sucrose content of 18 % (Fig. 2a). Further increase in the initial sugar content did not result in higher ethanol accumulation. Quality attributes, including total phenolic, anthocyanin, betacyanin and betaxanthin concentration and antioxidant activity of the wine, were improved with the increase in the initial sugar content up to 18 % and levelled off beyond this amount (Fig. 2b, Fig. 2c, Fig. 2d, Fig. 2e and Fig. 2f). A maximum value of (36.2±0.4) mg/mL, (10.6±0.4) mg/L, (66.5±0.8) mg/L, (64.3±0.6) mg/L and (64.1±0.9) % was obtained for phenolic, anthocyanin, betacyanin and betaxanthin concentrations and antioxidant activity, respectively, as measured by DPPH scavenging capacity. However, these results were not in agreement with those reported by Yuan *et al.* (*24*) that a higher amount of initial sugar (*e.g.* 24 %) is more favourable for ethanol accumulation. In contrast, Arroyo-Lopez *et al.* (*28*) reported that a sugar content higher than 20 % caused a decrease in the yeast cell growth rate, which may retard the ethanol production rate. In addition, wine fermentation with high sugar content negatively affected the production of volatile compounds, which might be detrimental to the quality of the wine (*29*). The high sucrose content might impose a high osmosis pressure on the yeast cells, reducing their performance during fermentation. From this study, it was concluded that an initial sucrose amount of 18 % (*m/V*) was the most favourable to obtain red dragon fruit wine with a high antioxidant profile.

#### Effect of initial pH

**Fig. 3:**
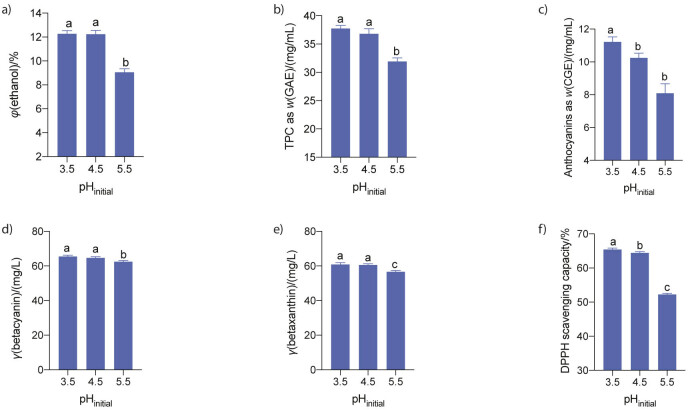
Effect of pH on: a) ethanol content, b) total phenolic, c) total anthocyanin, d) betacyanin, e) betaxanthin mass concentrations, and f) antioxidant activity of the red dragon fruit wine. The juice with different initial pH values and a sugar content of 18 % (*m*/*V*) was fermented by *S. cerevisiae* M7 at a pitching rate of 10^8^ CFU/mL and 25 °C for 7 days. Data are mean values of triplicate analyses±standard deviation. Mean values in each graph without a common letter differ significantly, p≤0.05. GAE=gallic acid equivalent, CGE=cyanidin-3-glucoside equivalent

The pH of the environment is another critical factor for the growth of yeast and wine fermentation (*28*), as it is known to alter the conformation, hence the function of the proteins embedded in the cell membrane and eventually affect the fermentation rate and the composition of the fermentation products (*30*). To investigate the effect of initial pH on the fermentation of red dragon fruit wine by *S. cerevisiae* M7, the pH of the juice was adjusted to 3.5, 4.5 and 5.5 before inoculation of the yeast. The results showed that ethanol productivity was approx. 12 % at the initial pH values of 3.5 and 4.5 (Fig. 3a). The total phenolic compounds, expressed as GAE, were approx. 37 mg/mL at initial pH=3.5 (Fig. 3b). Anthocyanin concentration was slightly higher at initial pH=3.5 than at pH=4.5 (Fig. 3c), while there was no significant difference in the concentrations of betacyanin and betaxanthin at these initial pH values (Fig. 3d and Fig. 3e). The antioxidant activity was approx. 65 % at the initial pH=3.5 (Fig. 3f). Increasing the initial pH of the juice to 5.5 significantly reduced ethanol production as well as the phenolic, anthocyanin and betalanin concentration and antioxidant activity of the wine (Fig. 3). The results of our study are consistent with those of Liu *et at*. (*30*), who reported that an initial pH=4.5 is most favourable for the growth and alcoholic fermentation of several *S. cerevisiae* strains, including Freddo, BH8 and N °.7303. However, it should be noted that the optimal pH for wine fermentation might depend on the strain, as *S. cerevisiae* NCIM 3045 achieved the highest ethanol yield in palm wine fermentation at pH=5.5 (*31*). Nevertheless, the results of the current study showed that the initial pH=4.5 was optimal for wine fermentation of red dragon fruit juice by *S. cerevisiae* M7 in terms of ethanol productivity, phenolic, betacyanin and betaxanthin concentration and antioxidant activity of the wine.

#### Effect of temperature

**Fig. 4:**
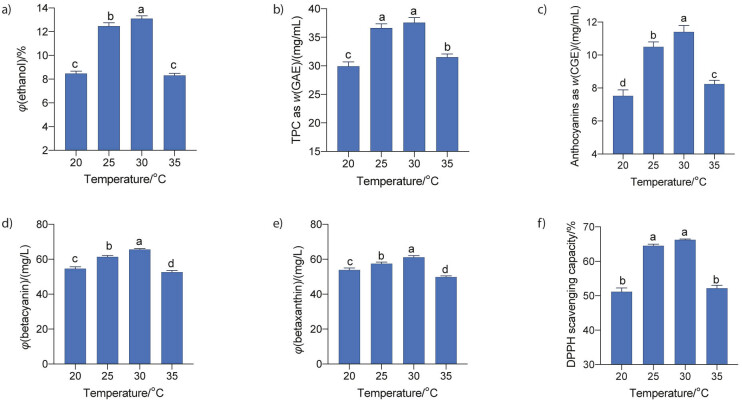
Effect of fermentation temperature on: a) ethanol content, b) total phenolic, c) total anthocyanin, d) betacyanin and e) betaxanthin mass concentrations, and f) antioxidant activity of the red dragon fruit wine. The fermentation temperature varied from 20 to 35 °C. The initial pH was 4.5, sugar content was 18 % (*m*/*V*) and *S. cerevisiae* M7 was inoculated at a pitching rate of 10^8^ CFU/mL with a fermentation time of 7 days. Data are mean values of triplicate analyses±standard deviation. Mean values in each graph without a common letter differ significantly (p≤0.05). GAE=gallic acid equivalent, CGE=cyanidin-3-glucoside equivalent

Culture temperature is an important factor affecting the physiology of microorganisms during fermentation. Consequently, the accumulation of fermented products in the culture is also affected (*26*). In this work, the strain *S. cerevisiae* M7 was used for the fermentation of red dragon fruit juice at different temperatures ranging from 20 to 35 °C, a pitching rate of 10^8^ CFU/mL, an addition of 18 % of sucrose and an initial pH=4.5. The results (Fig. 4) showed the highest volume fraction of alcohol (13.1±0.2) %, total phenolic compounds as GAE ((37.6±0.9) mg/mL), anthocyanin as CGE ((11.4±0.4) mg/L), betacyanin ((65.6±0.5) mg/L) and betaxanthin ((61.2±1.0) mg/L) at a fermentation temperature of 30 °C (Fig. 4a, Fig. 4b, Fig. 4c, Fig. 4d and Fig. 4e). There was no significant difference in antioxidant activities at 25 and 30 °C (Fig. 4f). The content of all these compounds in the culture was significantly lower at fermentation temperatures of 20 and 35 °C. These results are similar to those of Patil *et al.* (*32*), who reported an optimum temperature of 27.5 °C for the fermentation of sugarcane and papaya juice by *S. cerevisiae* (EC1118), while the optimum temperatures for the fermentation of palm juice (*31*) and pomegranate juice (*26*) were higher at 32 and 37 °C, respectively.

#### Effect of fermentation time

**Fig. 5:**
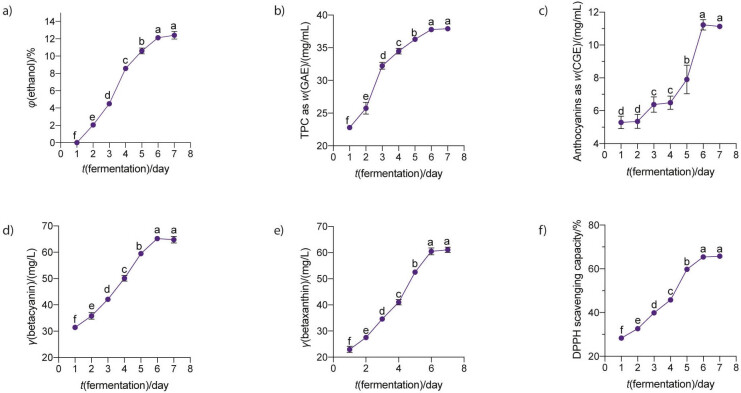
Changes in: a) ethanol content, b) total phenolic, c) total anthocyanin, d) betacyanin and e) betaxanthin mass concentration, and f) antioxidant activity of the red dragon fruit wine during fermentation. The juice with an initial pH=4.5 and a sugar content of 18 % (*m*/*V*) was fermented by *S. cerevisiae* M7 at 30 °C and a pitching rate of 10^8^ CFU/mL for different times (day). Data are mean value of triplicate analyses±standard deviation. Mean values in each graph without a common letter differ significantly, p≤0.05. GAE=gallic acid equivalent, CGE=cyanidin-3-glucoside equivalent

Fermentation time is critical for the quality of the wine and for obtaining a wine with a high content of bioactive compounds. In this study, the changes in ethanol production, phenolic compounds and antioxidants of red dragon wine during the first fermentation were analyzed. As shown in Fig. 5a, the ethanol volume fraction of the wine gradually increased from the first to the sixth day of fermentation to a maximum value of (12.1±0.2) %, while further fermentation beyond this time did not yield any additional amount of ethanol. In parallel with the increase in ethanol volume fraction, the concentrations of phenolics (as GAE), anthocyanin (as CGE), betacyanin, betaxanthin and the antioxidant activity of the wine also increased steadily during the first six days of fermentation and reached a maximum value of (37.8±0.4) mg/mL, (11.2±0.3) mg/L, (65.2±0.8) mg/L, (60.5±1.3) mg/L and (65.4±0.4) %, respectively (Fig. 5b, Fig. 5c, Fig. 5d, Fig. 5e and Fig. 5f). These results indicate that the primary fermentation period of six days was optimal for *S. cerevisiae* M7 to produce red dragon fruit wine. In other studies, Samson *et al.* (*26*) and Yuan *et al*. (*24*) reported that a fermentation time of 7 or 8 days was optimal for wine fermentation from pomegranate and green jujube juice, respectively. The differences in the optimal time for wine fermentation in the studies could be attributed to the differences in the yeast strains used, inoculum size, fermentation temperature and the type of juices, as these parameters greatly affect the growth and fermentation ability of the yeast strains.

## CONCLUSION

Our results show that the phenolic, anthocyanin, betacyanin and betaxanthin content and the antioxidant capacity of the wine produced from red dragon fruit juice pretreated at 70 °C significantly increased. Among the yeast isolates tested, *Saccharomyces cerevisiae* M7 had the highest values of these compounds in wine production. The optimum fermentation parameters for this strain were determined. These are a pitching rate of 10^8^ CFU/mL, an initial sugar content of 18 %, an initial pH=4.5, a temperature of 30 °C and a fermentation time of 6 days. Under these conditions, the amounts of ethanol, total phenols, anthocyanins, betacyanins, betaxanthins and antioxidant activity in this wine product were remarkably high. These results highlight the potential of red dragon fruit juice as a substrate for the production of high-quality wine with improved bioactive properties using the new strain *Saccharomyces cerevisiae* M7 as a starter.

## Appendix

**Table S1:** Screening of yeast strains for ethanol production

Yeast strain No.	*φ*(ethanol)/%	Yeast strain No.	*φ*(ethanol)/%
M1	-	M10	-
M2	3.97	M11	5.12
M3	-	M12	-
M4	-	M13	-
M5	-	M14	-
M6	-	M15	-
M7	5.16	M16	-
M8	-	M17	4.35
M9	-	M18	-

-no ethanol production
